# Functional social support and cognitive function in middle- and older-aged adults: a systematic review of cross-sectional and cohort studies

**DOI:** 10.1186/s13643-023-02251-z

**Published:** 2023-05-22

**Authors:** Lana Mogic, Emily C. Rutter, Suzanne L. Tyas, Colleen J. Maxwell, Megan E. O’Connell, Mark Oremus

**Affiliations:** 1 School of Public Health Sciences, Faculty of Health, University of Waterloo, 200 University Ave. W, Waterloo, ON N2L 3G1 Canada; 2 Department of Psychology, University of Saskatchewan, 9 Campus Drive, 154 Arts, Saskatoon, SK S7N 5A5 Canada

**Keywords:** Cognitive function, Functional social support, Systematic review

## Abstract

**Background:**

Intact cognitive function is crucial for healthy aging. Functional social support is thought to protect against cognitive decline. We conducted a systematic review to investigate the association between functional social support and cognitive function in middle- and older-aged adults.

**Methods:**

Articles were obtained from PubMed, PsycINFO, Sociological Abstracts, CINAHL, and Scopus. Eligible articles considered any form of functional social support and cognitive outcome. We narratively synthesized extracted data by following the Synthesis Without Meta-Analysis (SWiM) guidelines and assessed risk of bias using the Newcastle–Ottawa Scale (NOS).

**Results:**

Eighty-five articles with mostly low risk-of-bias were included in the review. In general, functional social support—particularly overall and emotional support—was associated with higher cognitive function in middle- and older-aged adults. However, these associations were not all statistically significant. Substantial heterogeneity existed in the types of exposures and outcomes evaluated in the articles, as well as in the specific tools used to measure exposures and outcomes.

**Conclusions:**

Our review highlights the role of functional social support in the preservation of healthy cognition in aging populations. This finding underscores the importance of maintaining substantive social connections in middle and later life.

**Systematic review registration:**

Rutter EC, Tyas SL, Maxwell CJ, Law J, O'Connell ME, Konnert CA, Oremus M. Association between functional social support and cognitive function in middle-aged and older adults: a protocol for a systematic review. BMJ Open;10(4):e037301. https://doi.org/10.1136/bmjopen-2020-037301

**Supplementary Information:**

The online version contains supplementary material available at 10.1186/s13643-023-02251-z.

## Background

Maintaining cognitive function is crucial for healthy aging [1–3]. Therefore, identifying and exploring modifiable risk or protective factors for cognitive function are key foci of aging research [4]. Social support is an important modifiable protective factor for cognitive function [5–8].

Structural social support is a quantifiable measure of social relationships, such as the number of people in one’s social network or the degree of participation in social events. Functional social support is the extent to which an individual perceives their needs can be met by members of their social network, such as the availability of someone to drive them to the doctor or help with grocery shopping, if required [9, 10].

Multiple reviews reported that large social networks and frequent engagement with these networks promote cognitive stimulation and protect against cognitive decline [11–14]. However, the literature has devoted less attention to functional social support and cognitive function, even though functional support more accurately represents the depth and quality of social support experienced by individuals than structural support [9].

Kelly et al. reviewed the association between functional social support and cognitive function in nine longitudinal studies of healthy older adults [15]. They reported variability in the direction and magnitude of the association, depending on the measures of functional support and cognitive function. Since Kelly et al.’s review [15], additional literature [6, 7, 16, 17] has emerged on the topic, underlining the need for an updated review.

We conducted this systematic review to investigate the association between functional social support and cognitive function across multiple cognitive domains (i.e., memory, executive function) and cognitive disease states (i.e., mild neurocognitive disorder, major neurocognitive disorder) in middle-aged and older adults. Our review focused exclusively on functional social support, reflecting Menec et al.’s conceptual distinction between objective (structural) and subjective (functional) social relationships: one may report many social contacts yet believe most will not help in times of need, or vice versa [18]. Importantly, this review differs from Costa-Cordella et al.’s recently published review [19], which included articles on structural and functional social support without age restrictions and excluded articles on neurological conditions characterized by cognitive deficits (e.g., mild or major neurocognitive disorder).

## Methods

Our review followed the 2020 Preferred Reporting Items for Systematic Reviews and Meta Analyses (PRISMA) guidelines [20] (Additional file 1). We departed slightly from our published protocol [4] and did not conduct a meta-analysis or formally assess publication bias, nor did we narratively synthesize the extracted data by sex, setting, or risk of bias level. These proposed undertakings were precluded by heterogeneity in definitions and measures of functional social support and cognitive function, as well as by multiple different means of reporting quantitative results in the included articles.

### Data sources and searches

We searched PubMed, PsycINFO, Sociological Abstracts, CINAHL and Scopus from inception to September 2021. Google Scholar was searched to retrieve grey literature. A medical librarian generated the syntax for PubMed (Additional file 2), which was adapted for the other databases.

### Eligibility criteria

The review included any study with a comparison group (e.g., cohort, cross-sectional, case–control) enrolling adults aged ≥ 40 years, regardless of residential setting (e.g., community, long-term care facility). Articles had to be published in English or French and report distinct results for persons in the age range of interest. The exposure was functional social support, sometimes called ‘perceived social support’ or ‘social support availability’, and the outcome was cognitive function. Included articles could assess global/overall functional social support or a subtype, such as emotional/informational support, tangible support, affectionate support, positive social interaction, using any tool or questionnaire. Similarly, the articles could measure cognitive function globally or by domain (e.g., memory, executive function) with any instrument or combination of tools (neuropsychological battery). We also included studies of neurological conditions characterized by cognitive deficits (e.g., mild or major neurocognitive disorder).

In line with the PICOS (population, intervention, comparator, outcome, and setting) framework, we present the inclusion criteria as follows:

- P = Adults aged 40 years or over from any residential setting, including those residing in the community or independent-living older age homes, or persons residing in institutionalized settings such as long-term care facilities;
- I = Any level of exposure to functional social support, defined broadly as one’s perception of the amount of help they would expect to receive from members of their social network in times of need;
- C = A different level of functional social support relative to ‘I’ above, e.g., comparing persons with lower scores on a social support scale (C) to persons with higher (better) scores on the scale (I);
- O = Any measure of differences between I and C, such as differences in cognition scale score or differences in the incidence or prevalence of a neurological condition; and
- S = Study conducted anywhere in the world and in any setting.

We excluded articles that did not assess any form of functional social support, cognitive function, or neurological condition with cognitive deficits. We also excluded articles that did not include comparison groups or articles published in languages other than English or French.

### Study selection, data extraction and risk of bias assessment

Following removal of duplicates, two reviewers used the eligibility criteria and Covidence software (Veritas Health Innovation, Melbourne, Australia) to independently screen the titles/abstracts and full texts of identified citations. Two reviewers independently extracted the following data from included articles into a prepared Excel spreadsheet: first author, year of publication, country of data collection, proportion female, setting, length of follow-up, type and measure of social support, type and measure of cognitive function, and outcomes. Reviewers extracted outcome data in the form reported by authors. Where possible, extracted data came from fully adjusted regression models. Two independent reviewers assessed risk of bias using the Newcastle–Ottawa Scale (NOS) [21]. In all cases, discrepancies between reviewers were resolved by consensus or a third reviewer.

### Synthesis methods

The extracted data were narratively synthesized in groups based on cognitive outcome, study design, and functional social support subtype. Studies of visuospatial skills or reasoning were classified under executive function; those of verbal memory, non-verbal memory, working memory, or episodic memory were classified under memory; and those of attention or processing speed were placed in their own unique category. We followed the Synthesis Without Meta-Analysis (SWiM) guidelines to conduct a narrative synthesis [22] and reported the effect measures contained in the included articles.

## Results

### Study characteristics

**Fig. 1:**
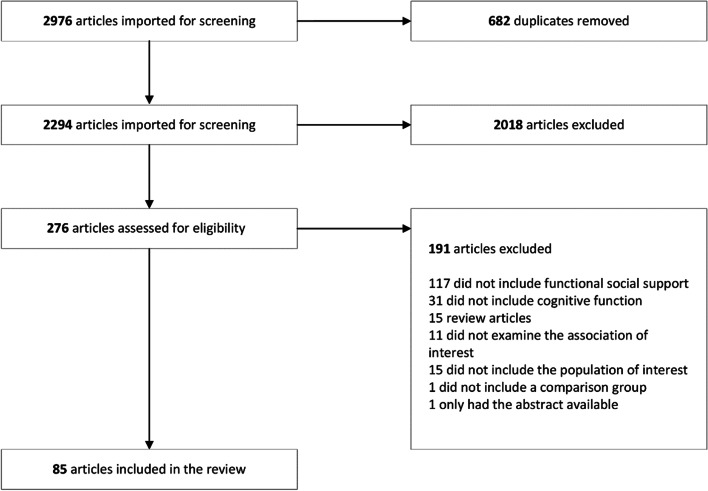
PRISMA Flow Diagram

**Fig. 2:**
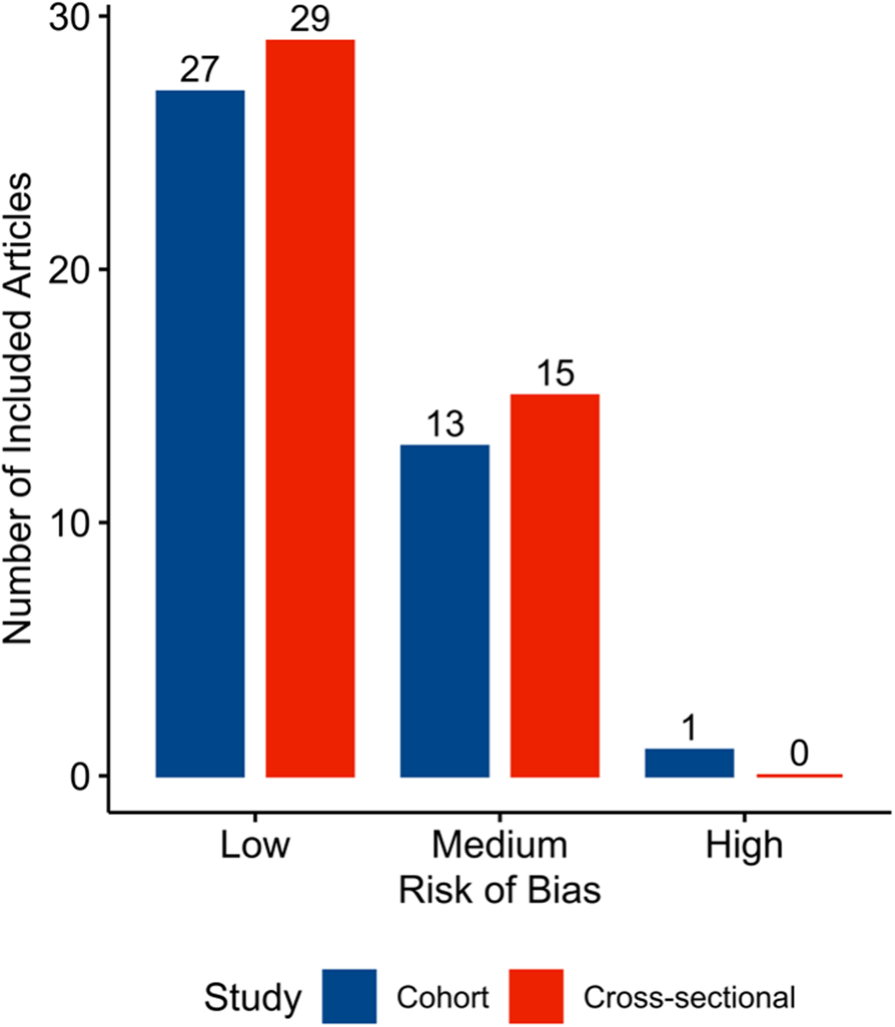
Risk of Bias

**Fig. 3:**
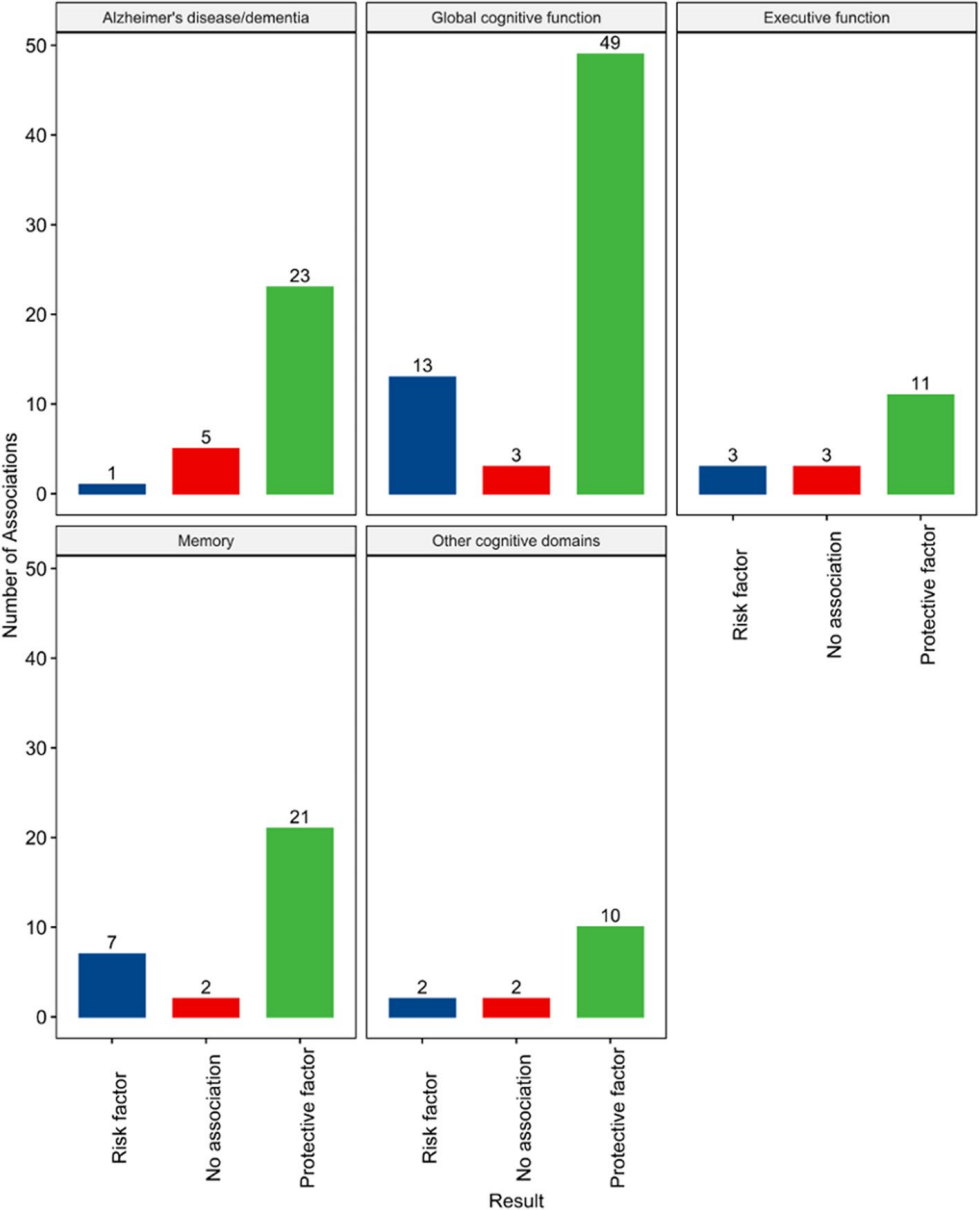
Count of Reported Associations between Functional Social Support and Cognition-related Outcomes in the Narrative Synthesis

Our search yielded 2,976 articles and 85 of these articles, published between 1986 and 2021, were included in the review (Fig. 1). Of these 85 articles, 44 were cross-sectional and 41 were cohort studies, with sample sizes ranging from 20 to 30,029 (Table 1). Most samples included community-dwelling persons, but four studies exclusively enrolled persons in institutionalized settings [23–26]. Nineteen articles examined dementia due to Alzheimer's disease (AD) or all-cause dementia, 38 examined global cognitive functioning or general cognitive impairment or decline, and 20 examined specific cognitive domains. Sixty-two articles reported multiple subtypes of functional social support. Common control variables were age, sex, race, education, income, social network, marital status, activities of daily living (ADLs), depression, and chronic conditions such as diabetes, cardiovascular disease, and hypertension. Most articles had low risk of bias (Table 2; Fig. 2). Overall, functional social support was protective against cognitive outcomes (Fig. 3).

**Table 1:** Study characteristics

**Author (Year)**	**Sample Size**	**Prop. Female**	**Age Range**	**Setting**	**Cognitive Outcome Measure**	**Analysis Method**	**Covariates**
**Cross-Sectional**							
Alpass et al., 2004 [27]	232	1.29 (3/232)	53.8 to 95.2	Community- dwelling	MMSE	Hierarchical regression analysis	Age, education, income, social network, depression
Bourgeois et al., 2020 [23]	359	15%	> 50	Institutionalized (HIV clinic)	MoCA	Poisson regression analysis	Age, sex, education, income, marital status, ethnicity, tobacco use, employment
Bourne et al., 2007 [28]	266	50 (134/266)	64	Community- dwelling	MHT (in 1947); Raven’s standard progressive matrices (at age 64)	Bivariate correlation	Sex
Conroy et al., 2010 [29]	802	53% (423/802)	65 to 102	Community- dwelling	AMT	Multinomial odds ratio	Education, social network, marital status, loneliness, depression
Deng & Liu, 2021 [30]	10,556	55.26%	≥ 65 years	Community- dwelling and institutionalized	Chinese-MMSE	Multivariate logistic regression	Age, sex, education, income, marital status, area of residence
Ficker et al., 2002 [24]	194	71.60%	NR	Institutionalized	MDRS	Independent t-test	Race, education
Frith & Loprinzi, 2017 [31]	1874	59.10%	60 to 85	Community- dwelling	WAIS: DSST	Weighted multivariable regression	Age, sex, race
Ge et al., 2017 [32]	3159	58.90%	60 to 105	Community- dwelling	Chinese-MMSE, EBMT, SDMT, Digit Span Backwards Test	Linear regression analysis	Education, income, social network, marital status, ADLs, depression
Gow et al., 2007 [33]	488	58%	NR	Community- dwelling	MHT—raw MHT scores converted into IQ scores	Regression analysis	Age, education, income, marital status, loneliness
Gow et al., 2013 [34]	1091	NR	NR (mean age 70)	Community- dwelling	WAIS-III UK and WMS-III UK, tests of reaction and inspection time	Ancova	Social network, marital status, loneliness, depression
Hamalainen et al., 2019 [35]	30, 029	50.90%	45 to 85	Community- dwelling	Mental Alternation Test, Animal Fluency test, Controlled Oral Word Association Test, Stroop test, RAVLT with immediate and 5-min recall	Multiple regression analysis	Age, sex, race, education, income
Harling et al., 2020 [36]	5059	53.6%	≥ 40	Community- dwelling	Battery from Health and Retirement study (orientation in time, episodic memory, number patterns)	Poisson regression; linear regression	Age, sex, education, income, marital status, country of origin, self-reported literacy, self-rated childhood health, father’s occupation, household size, employment status
Henderson et al., 1986 [37]	274	NR	70–79 80 +	Community- dwelling	GMS; MMSE	Mancova	Age, sex, marital status
Holtzman et al., 2004 [38]	354	68.60%	50 to 81	Community- dwelling	MMSE	Simultaneous linear or logistic Regression	Age, sex, race, education, depression
Jang et al., 2020 [39]	2061	66.8%	≥ 60	Community- dwelling	Korean-MMSE; self-rated cognitive health	Bivariate regression; hierarchical linear regression	Age, sex, education, social network, marital status, depression, chronic conditions (functional disability, chronic disease), tobacco use, alcohol use
Keller- Cohen et al., 2006 [40]	20	15/20	85–93	Independent living in retirement community	composite cognistat; BNT	Hierarchical multiple Regression; bivariate correlation	Education
Kim et al., 2019 [41]	410	252/410	60 +	Community- dwelling	VMS; CERAD-TS; MMSE	One-way ANCOVA	Age, sex, education, depression
Kotwal et al., 2016 [42]	3310	52%	62 to 90	Community- dwelling	MoCA-SA	Multiple linear regression	Age, sex, race, education, marital status, depression
Krueger et al., 2009 [43]	838	75(NR/883)	NR	Subsidized housing facilities and continuous care retirement communities	Episodic: Word List Memory, Recall, and Recognition; WMS; Semantic: BNT, National Adult Reading Test; Working: Digit Span Forward and Backward, Digit Ordering	Linear regression analysis	Age, sex, education, depression
La Fleur & Salthouse, 2017 [44]	2613	18–39: 66 40–59: 72 60–96: 63	NR	ND	Logical memory task; free recall task; paired associates’ task; Letter sets task; Shipley’s Abstraction; matrix reasoning	Multiple regression	Age, sex, education
Lee & Waite, 2018 [45]	2260	52.05	57–85	Community–dwelling	MoCA-SA	Multivariate regression	Age, sex, race, education
Mehrabi & Béland, 2021 [46]	1643	50.2%	≥ years	Community–dwelling	MoCA	Regression	Age, sex, education, income, smoking, alcohol consumption, sleeping disturbance
Millán-Calenti et al., 2013 [47]	579	57.2	≥ 65 years	Community–dwelling residents	MMSE; The Geriatric Depression Scale-Short Form	Multinomial logistic regression	Age, sex, education, ADLs
Murayama et al., 2019 [48]	897	50 (450/897)	≥ 65 years	Community- residents	MMSE-J	Multilevel logistic regression	Age, sex, education, income, social network, marital status
Nakamura et al., 2019 [49]	331	100 (331/331)	≥ 65 years	ND	BOMC	Unadjusted bivariate analysis	Age, education, ADLs, depression
Okabayashiet al., 2004 [50]	1976	NR	≥ 65 years	ND	Japanese-SPMSQ	Regression (unspecified)	Age, sex, education, depression
Oremus et al., 2019 [6]	21,241	51%	45–85	ND	RAVLT, Animal Fluency Test, Mental Alternation Test	Rao-Scott chi square	Age, region of residence, urban / rural residence, education
Oremus et al., 2020 [7]	21,241	51.3 (10,835/21241)	45–85	ND	RAVLT	Multiple linear regression	Age, sex, education, income, marital status, ADLs, depression
Pillemer & Holtzer, 2016 [51]	355	55.2 (196/355)	65.00- 95.00	Community- dwelling	RBANS	Linear regression analysis	Age, sex, education, depression
Poey et al., 2017 [52]	779	58 (452/779)	70–110 years	ND	Diagnosis of normal cognition, CIND, AD, and non-AD dementia	Multiple logistic regression	Age, sex, race, education, depression
Rashid et al., 2016 [53]	2005	68 (1363/2005)	60–99	Community- dwelling	ECAG	Regression Analyses	Age, sex, race, education, social network, marital status
Saenz et al., 2020 [54]	4,017 (married dyads)	50% (4017/8034)	50 +	Community- dwelling	Cross-Cultural Cognitive Examination	Regression analysis	Age, sex, education, income, ADLs, depression
Sims et al., 2014 [55]	175	45%	54–83	Community- dwelling	Stroop Color-Word Test, Judgment of Line Orientation; WAIS-R: The Block Design subscale, Digit Span Forward, Digit Span Backward; WMS: Logical Memory I and II Visual Reproductions I and II; The Grooved Pegboard, TMT	Multiple regression	Age, sex, race, education, depression
Weng et al., 2020 [56]	1706	53.01%	≥ 45 years	Community- dwelling	Subjective cognitive decline	Univariate and multiple logistic regression	Age, sex, race, education, marital status, depression, chronic conditions (coronary heart disease, diabetes), exercise, employment status
Yang et al., 2020 [57]	470	52.6%	≥ 65 years	Community- dwelling	CDR; MMSE	Multiple linear regression	Age, sex, education, income, ADLs, depression, functional assessment questionnaire, neuropsychiatric inventory questionnaire (nighttime behaviors, irritability, apathy, motor disturbances)
Yeh & Liu, 2003 [58]	4993	46.67% ( 2330/4989)	65 +	Community- dwelling	SPMSQ	Multiple regression analysis	Sex, education, marital status, loneliness, ADLs
Zahodne et al., 2014 [59]	482	54.1	55–85	Community- dwelling	NIH Toolbox Cognition module: Dimensional Change Card Sort, Flanker, List Sorting, Pattern Comparison, Picture Sequence Memory	Regression analysis	Race, education, loneliness
Zahodne et al., 2018 [60]	548	62.6	ND	Community- dwelling	NIH Toolbox Cognition module: Dimensional Change Card Sort, Flanker, List Sorting, Pattern Comparison, Picture Sequence Memory, Selective Reminding Test. Language scores, Benton Visual Retention Test, the Rosen Drawing Test, and the Identities and Oddities subtest of the DRW	Multiple regression	Race, education, income
Zank & Leipold, 2001 [61]	63	76%	53–96	Geriatric day care units	MMSE	Hierarchical regression analysis	Education, marital status
Zhaoyang et al., 2021 [62]	311	67%	70–90	Community- dwelling	5 cognitive domains (memory, executive function, attention, language, visual-spatial) with 10 neuropsychological instruments	Multilevel Poisson and logistic models	Age, sex, race, education, employment, marital status, living status
Zhu et al., 2012 [63]	120	37.50%	60–86	Community- dwelling	MMSE	Multiple regression analysis	Age, sex, education, income, social network, marital status,
Zuelsdorff et al., 2013 [64]	623	71%	40–73	Community- dwelling	RAVLT, Digits Forward, Digits Backward; WAIS-III: Letter-Number Sequence subtests; TMT, and Stroop Color-Word	Regression analysis	Age, sex, education, social network, marital status
Zuelsdorff et al., 2019 [65]	1052	69%	40–78	Community- dwelling	RAVLT; BVMT-R; WAIS-R: Logical Memory immediate and delayed recall subtests; TMT, Stroop; Color-Word Interference condition; WAIS: Digit Span Forward, Digit Span Backward, and Letter-Number Sequencing	Regression analysis	Age, sex, race, education, social network, marital status, ADLs
Zullo et al., 2021 [66]	1567	58.65%	≥ 65 years	Community- dwelling	Questionnaire de la Plaite Cognitive (QPC)	Binary logistic regression	Age, sex, depression, personality dimensions, quality of life, professional activity, interaction term between neuroticism and quality of life
**Cohort**							
Amieva et al., 2010 [67]	Study sample size = 3777, Analytic/included sample: 2089	59.9% (1251/2089)	ND	Community- dwelling	AD / Dementia diagnosis; MMSE; NINCDS-ADRDA criteria for AD	Multivariate analysis	Sex, education, social network, ADLs, Diabetes, CVD
Andel et al., 2012 [68]	10,106	52%	ND	Community- dwelling	Dementia diagnosis using DSM-4 criteria	Regression analysis	Age, sex, education, vascular disease
Bedard & Taler, 2020 [69]	11,152 (440 cases, 10,712 controls)	Controls: 55.3% Cases: 42.1 – 44.9%	45–85	NR	Animal Fluency Test, controlled oral word association test, mental alternation test, and Victoria Stroop test, Ray auditory verbal learning test, Miami prospective memory test	Binary logistic regression	Age, sex, education, marital status, depression, testing language
Bowling et al., 2016 [70]	9119	50.69% (4622/9119)	ND	Community- dwelling	Reading and comprehension test, arithmetic test, copying design test, general ability test	Multiple linear regression	Sex, education, social network, marital status
Camozzato et al., 2015 [71]	220	70%	ND	Community- dwelling	DSM5 and NINCDS-ADRDA criteria	Multivariate cox proportional- hazards moel	Age, sex, education, income, marital status, ADLs
Chen & Chang, 2016 [72]	2300	44.87%	65–93	Community- dwelling	SPMSQ; Chinese-MMSE	Multinomial logistic regression	Age, sex, education, ADLs, hypertension, diabetes, heart disease, stroke
Chen & Zhou, 2020 [73]	16, 786	NR	≥ 65 years	Community- dwelling	Chinese-MMSE	Generalized structural equation modelling (GSEM)	Age, sex, education, marital status, cardiometabolic diseases (diabetes, cardiovascular, stroke, heart disease), residence
Crooks et al., 2008 [25]	initial = 2249	100%	ND	Institutionalized	Telephone Interview for Cognitive Status; Telephone Dementia Questionnaire	Cox proportional hazards	Age, sex, education, social network, marital status, depression, stroke, myocardial infarction, diabetes, hypertension, PD
Dickinson et al., 2011 [74]	213	63.85%		Community- dwelling	CERAD; WMS-R; Logical Memory subtest; TMT, SDMT; WAIS-R: Digit Span Forward; ascending Digit Span task modeled after the Digit Ordering Test	Linear regression models	Age, sex, education, social network
Eisele et al., 2012 [75]	2367 (1869 = analytic sample)	65.90%	79–95	Community- dwelling	SIDAM	Multifactorial ANCOVA	Age, sex, education, marital status, ADLs, hypertension, CVD, coronary heart disease, alcohol use, BMI
Ellwardt et al., 2013 [76]	2255	54.00%	55–85	Community- dwelling	MMSE; coding task, and Reven's Colored Progressive Matrices	Latent growth mediation model	Age, sex, education, loneliness, ADLs
Heser et al., 2014 [77]	2300	ND	ND	Community- dwelling	SIDAM	Proportional hazard models, cox regression analysis	Age, sex, education, ADLs
Holtzman et al., 2004 [38]	354	68.60%	50–81	Community–dwelling	MMSE	Simultaneous linear regression	Age, sex, race, education, social network
Howrey et al., 2015 [78]	2767	58.29%	ND	Community- dwelling	MMSE	Multivariate analyses by using simultaneous linear or logistic regression	Age, sex, education, income, marital status, ADLs, hypertension, heart attack, stroke, diabetes, vision, Nativity, BMI
Hudetz et al., 2010 [26]	80	0%	55–85	Institutionalized	RBANS: Story Memory and Word List Memory subtests; BVMT-R	Stepwise multiple regression analysis	Age, sex, education, hypertension, hypercholesterolemia, angina, myocardial infarction, type 2 diabetes
Hughes et al., 2008 [79]	at baseline = 417, analytic = 217	51.80%	ND	Community- dwelling	MMSE; Stroop test, TMT, Hopkins verbal learning tests	Random effects model	Age, sex, education, social network, marital status
Kats et al., 2016 [80]	13,782	ND	48–64	Community- dwelling	DSST, DWRT, WFT	Generalized linear models	Age, sex, race, education, social network
Khondoker et al., 2017 [81]	10,055	46%	ND	Community- dwelling	The short-form IQCODE questionnaire and physician	Proportional hazard regression models	Age, sex, education, income, diabetes, CVD, stroke, hypertension, cancer
Khoo & Yang, 2020 [82]	1735	NR	40–70	NR	Brief Test of Adult Cognition by Telephone (BTACT)	Structural equation modelling	Age, sex, education, income, general health
Liao et al., 2018 [83]	6,863	29.20%	ND	Community- dwelling	Alice Heim 4-I test (AH4-I), an inductive reasoning test, and two tests of verbal fluency	Bivariate dual change score model; goodness of fit	Age, sex, race, education, income, marital status, coronary heart disease, stroke, diabetes, cancer, depressive symptoms
Liao & Scholes, 2017 [84]	10,241	53.30%	ND	Community- dwelling	Verbal fluency and letter cancellation task	Linear mixed model	Age, sex, education, income, ADLs
Liu et al., 2020 [85]	13, 636	55%	≥ 65 years	Community- dwelling	Dementia Scale (Degree of Independence in Daily Living for Older Adults with Dementia)	Multivariate adjusted Cox proportional hazards model	Age, sex, education, history of disease (stroke, hypertension, myocardial infarction, diabetes, cancer), smoking, alcohol drinking, BMI, time spent walking per day, psychological distress score, motor function score, social participation
Luo et al., 2021 [86]	497	48%	64–68	NR	Subtest of verbal comprehension index in German WAIS-R; verbal fluency and vocabulary; subtest of perceptual reasoning index in WAIS-R	Mplus8	NR
Miyaguni et al., 2021 [87]	15, 313	51.80%	≥ 65 years	Community- dwelling	I to IV and Medical, I (= 22 on MMSE), II (= 16), III (= 13), IV (= 6)	Multilevel survival analyses with sensitivity analyses model	Age, sex, education, marital status, depression, living conditions, present illness, smoking status, alcohol consumption, individual social support
Moreno et al., 2022 [88]	2242	100%	65–83	NR	Primary Mental Abilities Vocabulary Test; Category Fluency Test; Letter Fluency Test; Benton Visual Retention Test; California Verbal Learning Test; California Verbal Learning Test; Digit Span Test; Card Rotation Test	Linear mixed models with covariate adjustment	Age, race, education, income, region, job classification, major medical comorbidities
Murata et al., 2019 [16]	14,088	50.97%	65–99	Community- dwelling	Incident dementia ascertained upon eligibility for Japan’s public LTCI system, Level II or higher, on the index for the evaluation of care needs for people with dementia	Cox proportional hazard models	Age, sex, education, marital status, health behaviors (alcohol, smoking daily physical activity), cognitive complaints to predict dementia, depression
Noguchi et al., 2019 [89]	121 (analytic sample)	47.10%	ND	Community- dwelling	Japanese MoCA	Multivariable Linear regression analysis	Age, sex, income, ADLs, stroke, hypertension, dyslipidemia, diabetes, depression, living alone, BMI
Okely et al., 2021 [90]			70–84	Community- dwelling	5 questions about current state of participants’ memory	Spearman’s rho	Age, sex, education, depression, diabetes, cardiovascular disease, occupational social class, personality, living situation, anxiety, older age fluid cognitive ability
Pais et al., 2021 [91]	341	57.5%	60–85	Community- dwelling	MMSE	Multivariable Cox analysis of social support on cognitive impairment (hazard ratio)	Age, sex, social network, marital status,
Pillemer et al., 2019 [17]	493	57.20%	65–95	Community- dwelling	RBANS	Cox proportional hazard ratio	Sex, race, education, diabetes, chronic heart failure, arthritis, hypertension, depression, stroke, Parkinson’s disease, chronic obstructive lung disease, angina, myocardial infarction, depressive symptoms
Riddle et al., 2015 [92]	299	normal = 59.43%, MCI = 57.89%, dementia = 70.83%	ND	Community–dwelling	Neuropsychological battery to detect incident dementia or cognitive impairment	Χ2 for categorical variables and ANOVA, logistic regression models	Age, sex, race, education, ADLs, depression
Rote et al., 2021 [93]	2880	57.7%	≥ 65 years	Community- dwelling	MMSE	Logistic regression	Age, sex, country of birth (Mexico or USA), Medicaid (yes or no)
Saito et al., 2018 [94]	13,984	50.90%	ND	Community- dwelling	Long-term Care Insurance, The Degree of Autonomy in the Daily Lives of Elderly Individuals with Dementia Scale	Cox proportional hazard models	Age, sex, education, income, social network, marital status, ADLs, stroke, diabetes, depression, SCI, physical activity
Salinas et al., 2017 [95]	1834 (for dementia analysis)	44%		Community- dwelling	DSM-IV	Cox proportional hazard models	Age, sex, education, social network, marital status, atrial fibrillation, diabetes, CVD, smoking status, depression, physical activity, antihypertensive treatment
Seeman et al., 2001 [96]	1189	55.20%	70–79	Community- dwelling	BNT; WAIS-R	Multivariate linear regression	Age, sex, race, education, income, social network, marital status, physical activity
Sörman et al., 2015 [97]	1715	No Dementia: 53.3% all cause dementia: 65.1% AD: 73.9%		Community- dwelling	DSM-IV	Multivariate linear regression	Age, sex, education, CVD, stroke, HBP, diabetes, alcohol use, smoking status, obesity, stress, depression
Thomas & Umberson, 2018 [98]	2,788	64.70%	60–95	Community- dwelling	SPMSQ	Estimated growth curve models within a mixed-model framework Intercept (SE), Linear Slope	Age, sex, race, education, income, marital status, number of children, stressful life events
Wilson et al., 2015 [99]	529	78.90%		Institutionalized and community- dwelling	Clinical classification of MCI	Proportional hazards model	Age, sex, education, social network, loneliness, depression, negative life events
Yin et al., 2020 [100]	5897	51%	≥ 65 years	Community- dwelling	MMSE	Multivariable Cox regression (hazard ratio)	Age, sex, education, income / occupation, ADLS, residence, participation in physical activity, smoking, drinking, negative well-being, baseline MMSE, leisure activities, physical diseases
Zahodne et al., 2019 [101]	8,538	56.24%	45–93	Community- dwelling	Consortium to Establish a Registry for Alzheimer’s Disease Word List; Tests of semantic and letter fluency	Multivariate-adjusted standardized estimates	Age, sex, race, education, income, social network, heart disease, dyslipidemia, diabetes, nonlife threatening cancer, kidney failure, number of adults and children in childhood home, prenatal education, systolic BP, systemic inflammation, depression symptoms, perceived stress, BMI
Zahodne et al., 2021 [102]	578	663.5%	≥ 65 years	Community- dwelling	WHICAP neuropsychological battery (episodic memory, language, visuospatial functioning); NIH Toolbox cognition module (executive function, working memory)	Longitudinal models	Age, sex, race, education, depression, presence / absence of 15 chronic conditions, baseline cognition

*AMT* Abbreviated Mental Test, *BNT* Boston Naming Test, *BOMC* Blessed Memory Orientation Concentration Test, *BVMT-R* Brief Visuospatial Memory Test – Revised, *CERAD* Consortium to Establish a Registry for Alzheimer's Disease, *DRS* Dementia Rating Scale, *DSST* Digit Symbol Substitution Test, *DWRT* Delayed Word Recall Test, *EBMT* East Boston Memory Test, *ECAQ* Cognitive Assessment Questionnaire, *GMS* Geriatric Mental State, *MANCOVA* Multivariate analysis of Covariance, *MDRS* Mattis Dementia Rating Scale, *MHT* Moray House Test, *MMSE* Mini Mental State Examination, *MoCA* Montreal Cognitive, *RAVLT* Rey Auditory Verbal Learning Test, *RBANS* Repeatable Battery for the Assessment of Neuropsychological Status, *SCI* Subjective Cognitive Impairment, *SCOPA-COG* Scales for Outcomes in Parkinson’s Disease – Cognition, *SDMT* Symbol Digit Modalities Test, *SIDAM* Structured Interview for the Diagnosis of Dementia of the Alzheimer type, Multi-infarct Dementia and Dementia of other Aetiology, *SPMSQ* Short Portable Mental Status Questionnaire, *TMT* Trail Making Test A & B, *VMS* Verbal Memory Score, *WAIS* Wechsler Adult Intelligence Test, *WFT* Word Fluency Test, *WMS* Wechsler Memory Scale

**Table 2:** Overall risk of bias ratings

**Author, Year**	**Rating**	**Author, Year**	**Rating**
**Cross-Sectional Studies**			
Alpass et al., 2004 [27]	Medium	Millán-Calenti et al., 2013 [47]	Low
Bourgeois et al., 2020 [23]	Medium	Murayama et al., 2019 [48]	Low
Bourne et al., 2007 [28]	Medium	Nakamura et al., 2019 [49]	Low
Conroy et al., 2010 [29]	Medium	Okabayashi et al., 2004 [50]	Low
Deng & Liu, 2021 [30]	Medium	Oremus et al., 2019 [6]	Low
Ficker et al., 2002 [24]	Medium	Oremus et al., 2020 [7]	Low
Frith & Loprinzi, 2017 [31]	Low	Pillemer & Holtzer, 2016 [51]	Low
Ge et al., 2017 [32]	Low	Poey et al., 2017 [52]	Medium
Gow et al., 2007 [33]	Low	Rashid et al., 2016 [53]	Low
Gow et al., 2013 [34]	Low	Saenz et al., 2020 [54]	Low
Hamalainen et al., 2019 [35]	Low	Sims et al., 2014 [55]	Medium
Harling et al., 2020 [36]	Medium	Weng et al., 2020 [56]	Medium
Henderson et al., 1986 [37]	Medium	Yang et al., 2020 [57]	Low
Holtzman et al., 2004 [38]	Low	Yeh & Liu, 2003 [58]	Low
Jang et al., 2020 [39]	Low	Zahodne et al., 2014 [59]	Low
Keller-Cohen et al., 2006 [40]	Medium	Zahodne et al., 2018 [60]	Low
Kim et al., 2019 [41]	Low	Zank & Leipold, 2001 [61]	Low
Kotwal et al., 2016 [42]	Low	Zhaoyang et al., 2021 [62]	Low
Krueger et al., 2009 [43]	Medium	Zhu et al., 2012 [63]	Medium
La Fleur & Salthouse, 2017 [44]	Low	Zuelsdorff et al., 2013 [64]	Low
Lee & Waite, 2018 [45]	Low	Zuelsdorff et al., 2019 [65]	Low
Mehrabi & Béland, 2021 [46]	Low	Zullo et al., 2021 [66]	Medium
**Author, Year**	**Rating**	**Author, Year**	**Rating**
**Cohort Studies**			
Amieva et al., 2010 [67]	Low	Liu et al., 2020 [85]	Low
Andel et al., 2012 [68]	Low	Luo et al., 2021 [86]	Low
Bedard & Taler, 2020 [69]	Medium	Miyaguni et al., 2021 [87]	High
Bowling et al., 2016 [70]	Low	Moreno et al., 2022 [88]	Medium
Camozzato et al., 2015 [71]	Low	Murata et al., 2019 [16]	Low
Chen & Chang, 2016 [72]	Medium	Noguchi et al., 2019 [89]	Low
Chen & Zhou, 2020 [73]	Low	Okely et al., 2021 [90]	Medium
Crooks et al., 2008 [25]	Low	Pais et al., 2021 [91]	Low
Dickinson et al., 2011 [74]	Medium	Pillemer et al., 2019 [17]	Low
Eisele et al., 2012 [75]	Low	Riddle et al., 2015 [92]	Medium
Ellwardt et al., 2013 [76]	Low	Rote et al., 2021 [93]	Medium
Heser et al., 2014 [77]	Low	Saito et al., 2018 [94]	Low
Holtzman et al., 2004 [38]	Low	Salinas et al., 2017 [95]	Low
Howrey et al., 2015 [78]	Medium	Seeman et al., 2001 [96]	Low
Hudetz et al., 2010 [26]	Medium	Sörman et al., 2015 [97]	Low
Hughes et al., 2008 [79]	Low	Thomas & Umberson, 2018 [98]	Medium
Kats et al., 2016 [80]	Low	Wilson et al., 2015 [99]	Low
Khondoker et al., 2017 [81]	Low	Yin et al., 2020 [100]	Low
Khoo & Yang, 2020 [82]	Medium	Zahodne et al., 2019 [101]	Low
Liao & Scholes, 2017 [84]	Low	Zahodne et al., 2021 [102]	Low
Liao et al., 2018 [83]	Medium

### Narrative synthesis

#### Alzheimer’s disease or all-cause dementia

##### Cross-sectional studies

Four of the five cross-sectional studies reported on dementia, while the remaining study reported results for AD and non-AD dementia (Table 3). Four studies focused on functional social support, two of which reported no association with dementia. One found greater functional social support to be significantly associated with lower severity of dementia. One reported this support as being a moderate protective factor against AD, but a small risk factor for non-AD dementia. One study found that all-cause dementia was associated with lower satisfaction with diffuse social relationships, but not with close social relationships [29, 37, 42, 52, 57].

**Table 3:** Studies reporting outcome of Alzheimer’s Disease or dementia

**Author (year)**	**Dimension of FSS**	**Coefficient (CI or *P*-value)**	**Interpretation**
**Study Design: Cross-Sectional**			
Conroy et al., 2010 [29]	Perceived / Subjective	Dementia: OR = 1.0 (*p* = 0.934)	Perceived social support not associated with dementia
Kotwal et al., 2016 [42]	Perceived / Subjective	Dementia: 0.00 (-0.45, 0.46)	Perceived social support not associated with dementia
Poey et al., 2017 [52]	Perceived / Subjective	AD: RR = 0.567 (*p* = 0.174) Dementia: RR = 1.135 (*p* = 0.701)	Perceived social support has a protective effect against AD. Perceived support is associated with a small increased risk of non-AD dementia
Yang et al., 2020 [57]	Perceived / Subjective	Severity of dementia: x^2^ = 64.70 (*p* < 0.001)	Greater perceived social support significantly associated with lower severity of dementia
Henderson et al., 1986 [37]	Satisfaction with FSS	Dementia: 0.06 (*p* = 0.002)	Participants with dementia reported significantly lower satisfaction with diffuse social relationships than non-demented participants (value for satisfaction with close relationships not reported)
Study Design: Cohort			
Andel et al., 2012 [68]	Social Support at Work	AD: OR = 0.88 (0.76, 1.0) ** Dementia: OR = 0.87 (0.78, 0.97) **	Greater overall social support at work has protective effect against AD and dementia. Significant in case of dementia
Amieva et al., 2010 [67]	Satisfaction with FSS	AD: RR = 0.84 (0.3, 1.3) Dementia: RR = 0.77 (0.6, 0.9)	Satisfaction with social support has protective effect against dementia and AD; significant protective effect in case of dementia
Crooks et al., 2008 [25]	Satisfaction with FSS	Dementia: HR = 0.74 (0.78, 1.23)	Satisfaction with social support reduces risk of dementia
Camozzato et al., 2015 [71]	Perceived / Subjective	AD: HR = 0.19 (0.07, 0.52)^b^	Perceived support based on presence of confidants associated with significantly decreased risk of developing AD
Riddle et al., 2015 [92]	Perceived / Subjective	Dementia: x^2^ = 0.29 (*p* = 0.59)	Perceived support did not predict conversion to dementia
Heser et al., 2014 [77]	Emotional	AD: HR = 0.54 (0.19, 1.55) Dementia: HR = 1.02 (0.39, 2.66)	Small positive association between emotional support and all-cause dementia. Emotional support has protective effect against AD
Liu et al., 2020 [85]	Emotional	Dementia: HR = 1.10 (0.88, 1.37)	Receiving emotional social support associated with small (non-significant) increased risk of dementia
Miyaguni et al., 2021 [87]	Emotional	Dementia: 0.97 (0.94, 0.99)	Receiving emotional support significantly associated with decreased risk of dementia
Murata et al., 2019 [16]	Emotional	Dementia – Males: HR = 0.95 (0.39, 2.66) ^a^ Dementia – Females: HR = 0.98 (0.82, 1.18) ^a^	Emotional support has small protective effect against dementia in both males and females
Rote et al., 2021 [93]	Emotional	Low Support Likely dementia: 40.6% Increasing dementia: 49.1% No impairment: 10.3% High Support Likely dementia: 43.6% Increasing dementia: 36.9% No dementia: 19.5%	Values reported are conditional probabilities. Higher conditional probability of increasing dementia risk group belonging to low emotional support group
Saito et al., 2018 [94]	Emotional	Dementia: HR = 0.96 (0.89, 1.04) ^a^	Emotional support from family has small protective effect against dementia; effect even smaller in case of emotional support from friends. Small positive association between emotional support from relatives and dementia
Salinas et al., 2017 [95]	Emotional	Dementia: HR = 0.78 (0.56, 1.09)	Emotional support has protective effect against dementia
Sörman et al., 2015 [97]	Emotional	Dementia: HR = 0.82 (0.60, 1.11) AD: HR = 0.72 (0.48, 1.07)	Emotional support has protective effect against dementia and AD
Heser et al., 2014 [77]	Instrumental	Dementia: HR = 2.34 (0.91, 6.02) AD: HR = 3.57 (1.12, 11)	Large positive association between instrumental support and dementia and AD; association is significant in case of AD
Murata et al., 2019 [16]	Instrumental	Dementia: Female: 0.98^a^ (0.88, 1.09) Dementia: Male: 0.95^a^ (0.83, 1.08)	Instrumental support has a small protective effect against dementia in both males and females
Riddle et al., 2015 [92]	Instrumental	Dementia: x^2^ = 1.99 (*p* = 0.16)	Instrumental support did not predict conversion to dementia
Rote et al., 2021 [93]	Instrumental	Low Support Likely dementia: 40.0% Increasing dementia: 48.4% No impairment: 11.6% High Support Likely dementia: 43.7% Increasing dementia: 36.8% No dementia: 19.5%	Values reported are conditional probabilities. Higher conditional probability of increasing dementia risk group belonging to low instrumental support group
Khondoker et al., 2017 [81]	Positive social support	Dementia: HR = 0.87 (0.72, 1.06)	Positive social support has a small protective effect against dementia

*AD* Alzheimer's Disease, *CI* Confidence Interval, *FSS* Functional Social Support, *HR* Hazard Ratio, *OR* Odds Ratio, *RR* Relative Risk

^a^Effects merged using Borenstein: Murata et al. (2019) and Saito et al. (2018) both reported specific sources of functional social support (co-residing family, relatives, or friends), which were merged using Borenstein’s equation for reporting in the data tables (Borenstein et al., 2009)

^**^ Inverse of point estimate and confidence limits taken to convert outcome to yes versus no

##### Cohort studies

Nine of 14 cohort studies reported an outcome of all-cause dementia, four studies reported outcomes of AD and non-AD dementia independently, and one study reported an outcome of only AD (Table 3). Eight studies explored the effects of emotional social support, six of which found small to moderate protective effects against dementia (one reached statistical significance). One observed a small protective effect in both male and female strata. Two studies reported small positive, but not statistically significant, associations between emotional support and all-cause dementia. Two of the eight studies found moderate protective effects for emotional social support against AD [16, 25, 71, 77, 81, 85, 87, 92–95].

Four studies assessed instrumental social support, one of which reported a large positive association with both AD and non-AD dementia (statistically significant in the case of AD). Another study found small protective effects against dementia in both male and female participants. One study found that individuals identified as having increasing dementia were more likely to fall within the low instrumental support group. One study found no association [16, 77, 92, 93].

Two studies found satisfaction with social support to have moderate protective effects against dementia, with one being statistically significant. One of these also found satisfaction to have a moderate and nonsignificant protective effect against AD [25, 67]. Khondoker et al. reported positive social support had small protective effects against dementia [81]. Andel et al. showed workplace social support was protective against AD and non-AD dementia (statistically significant for non-AD) [68].

#### Global cognitive functioning

##### Cross-sectional studies

Three cross-sectional studies examined participant satisfaction with functional social support and global cognitive function (Table 4). Two reported positive yet statistically non-significant associations, and one found no association [27, 34, 40].

**Table 4:** Studies reporting outcome of global cognitive functioning

**Author (year)**	**Dimension of FSS**	**Coefficient (CI or *P*-value)**	**Interpretation**
**Study Design: Cross-Sectional**			
Alpass et al., 2004 [27]	Satisfaction with FSS	0.034 (*p*-value not reported)	Satisfaction with social support is positively but not significantly associated with cognitive function
Gow et al., 2013) [34]	Satisfaction with FSS	positive direction of association (*p* = 0.278)	Satisfaction with social support is positively but not significantly associated with cognitive function
Keller-Cohen et al., 2006 [40]	Satisfaction with FSS	Quantitative data for this variable not reported	Satisfaction with social relationships did not predict performance on Composite Cognistat or BNT
Bourgeois et al., 2020 [23]	Perceived / Subjective	1.72 (*p* = sig)	Perceived social support significantly positively associated with better outcome on MoCA
Conroy et al., 2010 [29]	Perceived / Subjective	OR = 1.3 (p = 0.175)	Low perceived social support (+ widowed and lives alone) positively associated with possible cognitive impairment
Ficker et al., 2002 [24]	Perceived / Subjective	3.589 (*p* < 0.001)	Cognitively impaired elders perceived their social support as significantly less adequate than did the cognitively intact participants
Krueger et al., 2009 [43]	Perceived / Subjective	0.068 (*p* = 0.003)^a^	Small significant positive association between perceived support and global cognitive function
Lee & Waite, 2018 [45]	Perceived / Subjective	Female—0.65 (*p* < 0.05) Male – no association	Significant positive effect of social support on cognition only in female participants. No association in male participants
Mehrabi & Béland, 2021 [46]	Perceived / Subjective	Partner—0.275 (0.028, 0.522) Children – no association Friends – no association Extended family – no association	Low perceived social support from partner significantly positively associated cognitive impairment. No association between perceived support from children, friends, or extended family and cognitive function
Oremus et al., 2019 [6]	Perceived / Subjective		Proportion of participants with low cognitive function greater among persons who reported low perceived social support (and vice versa)
Saenz et al., 2020 [54]	Perceived / Subjective (from spouse)	Husbands: 0.02 (0.01,0.03) Wives: 0.00 (-0.01, 0.01)	Perceived social support from wife significantly positively associated with the husband’s cognitive ability
Yeh & Liu, 2003 [58]	Perceived / Subjective (from friends)	0.11 (*p* = 0.005)	Perceived positive support from friends is significantly and positively associated with cognitive function
Zank & Leipold, 2001 [61]	Perceived / Subjective	R^2 =^ 0.085 (*p* < 0.05)	Perceived social support positively and significantly associated with cognitive function
Zhu et al., 2012 [63]	Perceived / Subjective	0.020 (*p* < 0.05)	Total perceived support positively and significantly associated with cognitive function
Zullo et al., 2021 [66]	Perceived / Subjective	OR = 0.93 (0.70, 1.24)	Individuals with subjective cognitive decline scored higher on the MSPSS indicating greater perceived social support
Deng & Liu, 2021 [30]	Emotional	Relatives/friends/neighbors: OR = 0.219 (0.154, 0.311) Children: OR = 0.400 (0.293, 0.546) Spouse: OR = 0.242 (0.160, 0.366)	Emotional support from relatives / friends / neighbors, children, or spouse significantly associated with a reduced risk of cognitive impairment
Harling et al., 2020 [36]	Emotional	0.72 (0.63, 0.82)	Emotional support significantly associated with decreased risk of cognitive impairment
Kim et al., 2019 [41]	Emotional	4.160 (*p* = 0.002)	Emotional support significantly positively associated with cognitive function
Murayama et al., 2019 [48]	Emotional	Male: OR = 0.46 (0.24, 0.86) ** Female: OR = 0.59 (0.35, 0.99) **	Higher emotional support significantly associated with decreased risk of cognitive impairment
Nakamura et al., 2019 [49]	Emotional	-0.02 (*p* = 0.04)	Higher emotional social support significantly associated with better cognitive scores
Okabayashi et al., 2004 [50]	Emotional	Spouse: 0.02 (*p* < 0.05) Children: 0.05 (*p* < 0.05) Others: 0.01 (*p* < 0.05)	Emotional support from spouse, children, or others all significantly positively associated with cognitive function
Pillemer & Holtzer, 2016 [51]	Emotional	1.620 (0.343, 2.897)	Emotional support positively associated with cognitive function
Weng et al., 2020 [56]	Emotional	OR = 1.68 (1.37 to 2.06)	Insufficient emotional support significantly associated with increased reporting of subjective cognitive decline
Deng & Liu, 2021 [30]	Instrumental	OR = 0.242 (0.630, 0.804)	Instrumental (financial) support significantly associated with decreased risk of cognitive impairment
Harling et al., 2020 [36]	Instrumental	0.73 (0.64, 0.82)	Instrumental support significantly associated with decreased risk of cognitive impairment
Millán-Calenti et al., 2013 [47]	Instrumental	OR = 1.04 (0.27, 4.0) ^b^	Small positive association between instrumental support and cognitive function
Murayama et al., 2019 [48]	Instrumental	Male: OR = 0.43 (0.22, 0.83) ^b^ Female: OR = 0.62 (0.30, 1.28) ^b^	Higher instrumental support associated with decreased risk of cognitive impairment. Significant association in males
Nakamura et al., 2019 [49]	Instrumental	0.00 (*p* = 0.97)	No association between instrumental support and cognitive function
Pillemer & Holtzer, 2016 [51]	Instrumental	-0.235 (-1.535, 1.066)	Tangible support has a small negative association with cognitive function
Ge et al., 2017 [32]	Emotional + Instrumental	R^2 =^ 0.11 (*p* < 0.001)	Emotional and instrumental support significantly positively associated with cognitive function
Gow et al., 2007 [33]	Emotional + Instrumental	0.14 (*p* < 0.01)	Emotional and instrumental support significantly positively associated with IQ
Holtzman et al., 2004 [38]	Emotional + Instrumental	0.25 (*p* < 0.0005)	Emotional and instrumental support significantly positively associated with cognitive function
Pillemer & Holtzer. 2016 [51]	Positive Interaction	B = 1.8883 (0.595, 3.171)	Positive social interaction positively associated with cognitive function
Pillemer & Holtzer, 2016 [51]	Affectionate	B = -0.093 (-1.369, 1.183)	Affectionate social interaction not associated with cognitive function
Rashid et al., 2016 [53]	FSS	OR = 2.6 (1.2–5.4)	Increased risk of cognitive impairment among individuals with poor social support
Jang et al., 2020 [39]	Family Solidarity	0.00	No association between family solidarity and cognitive function
**Study Design: Cohort**			
Hughes et al., 2008 [79]	Satisfaction with FSS	0.09 (*p* = 0.22)	Positive association between satisfaction with social support and cognitive function
Bowling et al., 2016 [70]	Perceived / Subjective	Family: -0.01 (-0.30, 0.27) Friend: 0.02 (-0.29, 0.32)	Small negative association between perceived support from family and cognitive function. Small positive association between perceived support from friends and cognitive function
Chen & Zhou, 2020 [73]	Perceived / Subjective	OR = 2.09 (*p* < 0.001)	Social isolation significantly associated with cognitive impairment
Eisele et al., 2012 [75]	Perceived / Subjective	F-ratio = 2.114	Positive association between perceived support and cognitive function
Howrey et al., 2015 [78]	Perceived / Subjective	Rapid decline: 1.89 (*p* < 0.001) Slow decline: 0.25 Stable: 0.35	In rapid decline group, social support significantly associated with increases in MMSE
Hudetz et al., 2010 [26]	Perceived / Subjective	0.01 (*p* = 0.64)	Small positive association between perceived support and cognitive function
Kats et al., 2016 [80]	Perceived / Subjective	African Americans: -0.01 (-0.14, 0.12); Caucasians: 0.01 (-0.05, 0.05)	Small negative association between perceived support and cognitive function in African American population. Small positive association between perceived support and cognitive function in Caucasian population
Luo et al., 2021 [86]	Perceived / Subjective	b = 1.90 (*p* = 0.050)	Quality of social relationships significantly predicts cognitive function
Moreno et al., 2022 [88]	Perceived / Subjective	0.066 (*p* < 0.001)	Significant positive association between perceived social support and cognitive function
Pais et al., 2021 [91]	Perceived / Subjective (from friends)	HR = 0.77 (0.635, 0.933)	Perceived social support from friends significantly associated with a reduced risk of cognitive impairment
Bedard & Taler, 2020 [69]	Emotional	OR = 0.97 (0.95, 0.99)	Emotional support had a small but significant protective effect against cognitive decline
Chen & Chang, 2016 [72]	Emotional	Starting high and declining: 0.87 (0.71, 1.07) Starting low and declining: 0.77 (0.60, 0.99)	Emotional social support had a significant protective effect in the starting low and declining group compared with the high-stable group. (Protective but not statistically significant effect in starting high and declining group)
Ellwardt et al., 2013 [76]	Emotional	0.03 (intercept), 0.40 (slope), *p* = 0.06	Emotional support positively associated with cognitive function
Holtzman (2004) [38]	Emotional	Continuous model: 0.15 (*p* < 0.005) Categorical model: 0.18 (*p* < 0.004)	Emotional support was a significant predictor of MMSE scores
Hughes et al., 2008 [79]	Emotional	-0.05 (*p* = 0.45)	Small negative association between emotional support and cognitive function
Noguchi et al., 2019 [89]	Emotional	-0.42 (*p* = 0.462)	Emotional support negatively associated with cognitive function
Pillemer et al., 2019 [17]	Emotional	Incident cognitive decline: HR = 1.43 (0.94,2.18) Cognitive decline – males: HR = 1.62 (0.93,2.84) Cognitive decline – females: HR = 1.39 (0.68,2.84)	Emotional support positively associated with cognitive decline
Seeman et al., 2001 [96]	Emotional	1.26 (*p* = 0.07)	Emotional support positively associated with cognitive function
Thomas & Umberson, 2018 [98]	Emotional (from children)	-0.004, *p* < 0.05	Support from children related to fewer cognitive limitations
Bedard & Taler, 2020 [69]	Instrumental	OR = 0.98 (0.94, 1.02)	Instrumental support had a small protective effect against cognitive decline
Dickinson et al., 2011 [74]	Instrumental	0.578 (*p* = 0.0333)	Instrumental support significantly positively associated with cognitive function
Ellwardt et al., 2013 [76]	Instrumental	-0.01 (intercept), -0.02 (slope)	Small negative association between instrumental support and cognitive function
Hughes et al., 2008 [79]	Instrumental	0.01 (*p* = 0.88)	Small positive association between instrumental support and cognitive function
Noguchi et al., 2019 [89]	Instrumental	0.38 (*p* = 0.642)	Instrumental support positively associated with cognitive function
Pillemer et al., 2019 [17]	Instrumental	Incident cognitive decline: HR = 1.75 (1.12,2.72) Cognitive decline – males: HR = 1.91 (1.00,3.62) Cognitive decline – females: HR = 1.78 (0.94,3.35)	Instrumental support positively associated with cognitive decline
Seeman et al., 2001 [96]	Instrumental	-0.04 (*p* = 0.93)	Small negative association between instrumental support and cognitive function
Yin et al., 2020 [100]	Instrumental (sick care)	HR = 0.795 (0.550, 1.148)	Instrumental support negatively associated with cognitive impairment
Noguchi et al., 2019 [89]	Emotional + Instrumental	Co-residing family: 0.28, *p* = 0.813 Non-residing family and relatives: 0.51 (*p* = 0.283) Neighbours and friends: 1.23, *p* = 0.006	Significant positive association between emotional and instrumental social support from neighbours and friends and MoCA-J scores. Negative association between emotional and instrumental support from co-residing family or non-residing family and relatives and cognitive function

*CI* Confidence Interval, *FSS* Functional Social Support, *HR* Hazard Ratio, *MoCA-J* Japanese version of the Montreal Cognitive Assessment, *OR* Odds Ratio, *RR* Relative Risk

^a^Effects merged using Borenstein (Borenstein et al., 2009)

^b^Inverse of point estimate and confidence limits taken to convert outcome to yes vs. no or high vs. low

Twelve cross-sectional studies explored the association between perceived or subjective functional social support and global cognitive function, with 11 reporting positive associations (10 statistically significant), and one reporting a negative association (Table 4). One study observed significant positive effects among females only. One reported that support from a wife was positively associated with a husband’s cognitive function, but not vice versa. One observed a positive association for spouse-provided support, but not support from children, friends, and extended family. One found links between greater subjective cognitive decline and greater levels of perceived social support [6, 23, 24, 29, 43, 45, 46, 54, 58, 61, 63, 66].

Eight studies assessed the association between emotional social support and global cognitive function; authors reported positive associations in all eight, with seven reaching statistical significance. Six studies explored the effect of instrumental social support on cognitive function and two found statistically significant positive associations, one found a non-significant positive association, one found no association, one reported a small (non-significant) negative association, and one found positive associations in male (significant) and female (non-significant) strata. Three studies assessed the combined effects of emotional and instrumental social support on global cognitive function and found significant positive associations [30, 32, 33, 36, 38, 41, 47–51, 56].

Rashid et al. assessed general functional social support and observed that individuals with lower reported levels of support were at an increased risk of cognitive impairment [53]. Jang et al. used family solidarity as a measure of functional social support and found no association between this variable and cognitive function [39].

##### Cohort studies

One study found a positive association between functional social support and global cognitive function. Nine other studies assessed the association between perceived / subjective social support and global cognitive function, with six reporting positive associations, four of which were significant. One reported a negative association for Black people and a positive association for White people, although neither was significant. One showed a negative association for support from the family and a positive association for support from friends, with neither being statistically significant. One found perceived social support to be significant positively associated with cognitive function in persons whose cognition test scores were rapidly declining but found no association when scores were slowly declining or stable [26, 70, 73, 75, 78–80, 86, 88, 91].

Nine other cohort studies assessed the impact of emotional social support on global cognitive function. Three reported positive associations, one of which was significant. Two studies reported negative associations, neither of which was significant. In one study, emotional social support received from participants’ children was inversely associated with cognitive function. Similarly, inverse associations were found in male and female strata, though neither was statistically significant. One study identified significant protective effects for emotional support in persons whose baseline cognition was low and declining over time, and non-significant protective effects in those with high and declining cognition, compared to individuals with high and stable cognition [17, 38, 69, 72, 76, 79, 89, 96, 98].

Eight cohort studies explored instrumental social support and global cognitive function. Six studies reported positive associations, one of which was statistically significant. Three found non-significant negative associations. One study assessed the combined effects of emotional and instrumental social support, stratified by the source of support (co-residing family, non-residing family and relatives, neighbours and friends), and reported significant positive associations in the neighbours and friends stratum; the associations in the other two strata were inverse and non-significant [17, 69, 74, 76, 79, 89, 96, 100].

#### Studies reporting outcomes by cognitive domain

Twenty-seven studies examined the effects of functional social support on one or more specific cognitive domains (Table 5). Most studies assessed multiple domains, with 17 studies examining memory, 13 executive function, 3 attention and processing speed, 4 language ability, and 3 mild cognitive impairment (MCI).

**Table 5:** Studies Reporting other cognitive outcomes

**Author (year)**	**Dimension of FSS**	**Coefficient (CI or *P*-value)**	**Interpretation**
**Study Design: Cross-Sectional, Outcome: Executive Function**			
Gow et al., 2013 [34]	Satisfaction with FSS	positive direction of association; *p* = 0.075	Satisfaction with social support is positively but not significantly associated with executive function
Bourne et al., 2007) [28]	Emotional	-0.14 (*p* < 0.05)	Emotional support significantly negatively associated with executive function
Frith & Loprinzi, 2017 [31]	Emotional	Any support:* B* = 6.4 (2.9, 10)	Emotional functional social support significantly positively associated with executive function (of individual support types, only spousal support significantly associated with cognition)
La Fleur & Salthouse, 2017 [44]	Emotional	0.10 (*p* < 0.001)	Emotional support significantly positively associated with executive function
Zahodne et al., 2014 [59]	Emotional	0.17 (0.06) 0.09 (0.06)	Emotional support positively associated with executive function
Bourne et al., 2007 [28]	Instrumental	-0.13 (*p* < 0.05)	Satisfaction with instrumental support negatively associated with executive function
La Fleur & Salthouse, 2017 [44]	Instrumental	0.02 (*p* > 0.01)	Small positive association between instrumental support and executive function
Zahodne et al., 2014 [59]	Instrumental	DCCS: -0.04 (0.05) Flanker: 0.00 (0.05)	Instrumental support not associated with executive function
Ge et al., 2017 [32]	Emotional + Instrumental	R^2 = 1.44 (*p* < 0.001)	Emotional and instrumental support significantly positively associated with executive function
Hamalainen et al., 2019 [35]	Perceived / Subjective	B = 0.002 (*p* = 0.001)	Small positive association between perceived support and executive function
Krueger et al., 2009 [43]	Perceived / Subjective	0.089 (*p* = 0.036) ^a^	Perceived support significantly positively associated with executive function
**Study Design: Cohort, Outcome: Executive Function**			
Dickinson et al., 2011 [74]	Instrumental	0.284 (*p* = 0.0064) 0.578 (*p* = 0.0333)	Instrumental support significantly positively associated with executive function
Liao & Scholes, 2017 [84]	Positive social support	0.017 (0.009, 0.026)	Positive social support significantly positively associated with executive function
Liao et al., 2018 [83]	Confiding support	Y = − 0.05 (− 0.17, 0.07)	No association between confiding support and executive function
Hudetz et al., 2010 [26]	Perceived / Subjective	z-score = -0.01, *p* = 0.33	Perceived social support does not significantly predict post-operative executive functioning
Zahodne et al., 2021 [102]	Emotional	0.11 (not significant)	Emotional social support positively associated with executive function
Zahodne et al., 2021[102]	Instrumental	-0.03 (not significant)	Instrumental social support negatively associated with executive function
**Study Design: Cross-Sectional, Outcome: Memory**			
Gow et al., 2013 [34]	Satisfaction with FSS	positive direction of association (*p* = 0.275)	Satisfaction with social support is positively but not significantly associated with memory
Ge et al., 2017 [32]	Emotional + Instrumental	Working: R^2^ = 0.18 (*p* < 0.05) Episodic: R^2^ = 0.11 (*p* < 0.001)	Emotional and instrumental support significantly positively associated with both episodic and working memory
Hamalainen et al., 2019 [35]	Perceived / Subjective	B = 0.002 (*p* < 0.001)	Small positive and significant association between perceived support and memory
Krueger et al., 2009 [43]	Perceived / Subjective	Episodic: 0.023 (*p* = 0.444) Semantic: 0.055 (*p* = 0.056) Working: 1.07 (*p* = 0.003)	Small positive association between perceived support and episodic and semantic memory. Much larger and statistically significant positive association between perceived support and working memory
Okely et al., 2021 [90]	Perceived / Subjective	- 0.169 (*p* < 0.05)	Lower perceived social support significantly associated with increased memory problems
Zuelsdorff et al., 2013 [64]	Perceived / Subjective	Immediate: 0.006 (not significant) Verbal:0.037 (not significant) Working: -0.024 (not significant)	Small positive association between perceived support and immediate and verbal memory. Small negative association between perceived support and working memory
Zuelsdorff et al., 2019 [65]	Perceived / Subjective	Immediate: 0.07 (*p* = 0.01) Verbal: 0.04 (not significant) Working: 0.04 (not significant) Visual: 0.09 (*p* < 0.001)	Perceived support significantly positively associated with immediate and visual memory. Perceived support positively associated with verbal and working memory
Kim et al., 2019 [41]	Emotional	1.696 (*p* = 0.003)	Higher emotional support significantly associated with better verbal memory
La Fleur & Salthouse, 2017 [44]	Emotional	0.11 (*p* < 0.001)	Emotional support significantly positively associated with memory
Oremus et al., 2020 [7]	Emotional	Immediate: B = 0.06 (0.03, 0.09) Delayed: B = 0.05 (0.02, 0.08)	Emotional support significantly positively associated with both immediate and delayed memory
Zahodne et al., 2014 [59]	Emotional	Working: 0.09 Episodic: 0.09	Emotional support positively associated with both working and episodic memory
La Fleur & Salthouse, 2017 [44]	Instrumental	-0.01 (*p* > 0.01)	No association or small negative association between instrumental support and memory
Sims et al., 2014 [55]	Instrumental	-0.17 (*p* < 0.05)	Significant negative association between instrumental support and nonverbal recall
Zahodne et al., 2014 [59]	Instrumental	Working: 0.01 Episodic: -0.01	Small positive association between instrumental support and both working memory. Small negative association between instrumental suport and episodic memory
Oremus et al., 2020 [7]	Positive	Immediate: B = 0.05 (0.02, 0.07) Delayed: B = 0.04 (0.01, 0.07)	Positive support significantly positively associated with both immediate and delayed recall
Oremus et al., 2020 [7]	Affectionate	Immediate: B = 0.05 (0.02, 0.08) Delayed: B = 0.05 (0.02, 0.07)	Affectionate support significantly positively associated with both immediate and delayed recall
**Study Design: Cohort, Outcome: Memory**			
Hudetz et al., 2010 [26]	Perceived / Subjective	z-score = -0.02, *p* = 0.40	Perceived social support does not significantly predict post-operative verbal memory
Zahodne et al., 2018 [60]	Perceived / Subjective	Working: R^2 = 0.18 (*p* < 0.05) Episodic: R^2 = 0.11 (*p* < 0.001)	Significant positive association between perceived social support and both working and episodic memory
Hughes et al., 2008 [79]	Emotional	-0.02 (*p* = 0.83)	Small negative association between emotional support and memory
Zahodne et al., 2021 [102]	Emotional	Working: 0.04 (not significant) Episodic: -0.11 (not significant)	Small positive association between emotional support and working memory. Negative association between emotional support and episodic memory
Hughes et al., 2008 [79]	Instrumental	0.01 (*p* = 0.93)	Small positive association between instrumental support and memory
Zahodne et al., 2021 [102]	Instrumental	Working: -0.03 (not significant) Episodic: 0.00 (not significant)	Small negative association between instrumental support and working memory. No association between instrumental support and episodic memory
Hughes et al., 2008 [79]	Satisfaction with FSS	0.18 (*p* = 0.06)	Satisfaction with social support positively associated with memory
Liao & Scholes, 2017 [84]	Positive social support	0.018 (0.003, 0.033)	Positive social support significantly positively associated with memory
**Study Design: Cross-Sectional, Outcome: Language**			
La Fleur & Salthouse, 2017 [44]	Emotional	0.13 (*p* < 0.001)	Emotional support significantly positively associated with language ability
La Fleur & Salthouse, 2017 [44]	Instrumental	0.01 (*p* > 0.01)	No association or small positive association between instrumental support and language ability
**Study Design: Cohort, Outcome: Language**			
Hudetz et al., 2010 [26]	Perceived / Subjective	z-score = 0.01 (*p* = 0.69)	Perceived social support does not significantly predict verbal memory
Zahodne et al., 2018 [60]	Perceived / subjective	Initial cognitive level: 0.022 (-0.010, 0.054) Annual rate of cognitive change: 0.029 (-0.035, 0.092)	Reported childhood social support positively but not significantly associated with initial verbal fluency and rate of decline in verbal fluency
Zahodne et al., 2021 [102]	Emotional	-0.05 (not significant)	Negative association between emotional support and language ability
Zahodne et al., 2021 [102]	Instrumental	-0.07 (not significant)	Negative association between instrumental support and language ability
**Study Design: Cross-Sectional, Outcome: MCI**			
Kotwal et al., 2016 [42]	Perceived / Subjective	0.02 (-0.33,0.37)	Perceived social support positively associated with better outcome on MoCA-SA
Poey et al., 2017 [52]	Perceived / Subjective	RRR = 0.962 (*p* = 0.259) (reference group no social support available)	Social support has a slightly protective effect on the onset of MCI
Zhaoyang et al., 2021 [62]	General social support	-0.13 (-0.34, 0.07)	Negative association between general social support and MCI
**Study Design: Cohort, Outcome: MCI**			
Wilson et al., 2015 [99]	Negative social interaction	HR = 1.09 (0.81, 1.495)^a^	Negative social interaction positively associated with MCI
**Study Design: Cross-Sectional, Outcome: Attention / Processing Speed**			
Zuelsdorff et al., 2013 [64]	Perceived / Subjective	0.084 (*p* < 0.05)	Perceived social support significantly positively associated with processing speed
Zuelsdorff et al., 2019 [65]	Perceived / Subjective	0.05 (not significant – specific *p* value not reported)	Perceived social support positively associated with processing speed
**Study Design: Cohort, Outcome: Attention / Processing Speed**			
Hughes et al., 2008 [79]	Emotional	0.07 (*p* = 0.95)	Small positive association between emotional support and attention / processing speed
Hughes et al., 2008 [79]	Instrumental	-0.004 (*p* = 0.99)	Instrumental support not associated with attention / processing speed
Hughes et al., 2008 [79]	Satisfaction with FSS	1.24 (*p* = 0.30)	Satisfaction with social support positively associated with attention / processing speed

*CI* Confidence Interval, *FSS* Functional Social Support, *HR* Hazard Ratio, *MCI* Minor Neurocognitive Disorder, *MoCA-SA* Montreal Cognitive Assessment Survey Adaptation, *RR* Relative Risk

^a^Effects merged using Borenstein (Borenstein et al., 2009)

##### Memory

*Cross-Sectional Studies*. Ten cross-sectional studies explored the association between functional social support and memory. One found a positive, non-significant association for satisfaction with available support. Two of five studies reported positive and statistically significant associations between perceived social support and memory. Two reported positive associations between perceived support and verbal memory, with the only statistically significant association involving memory measured longitudinally. They also found negative and non-significant associations between perceived support and working memory at both time periods, and a positive and significant association between perceived support and visual memory measured longitudinally. One found a significant association between lower perceived social support and greater problems with memory or forgetfulness [34, 35, 43, 64, 65, 90].

Four studies examining emotional social support and memory reported positive associations, with results in three achieving statistical significance. One found the association between emotional support and verbal memory to be mediated by hippocampal volume, one reported similar strengths of association for immediate and delayed recall memory, and one found positive associations of the same magnitude for working and episodic memory [7, 41, 44, 59].

Three studies assessed the effects of instrumental social support on memory: one reported a statistically significant negative association with general memory [55]; one found a small and non-significant negative association with overall memory [44]; and one identified a small positive and non-significant association with working memory and a small negative and non-significant association with episodic memory [59]. Finally, Oremus et al. found positive social interactions and affectionate support to be independently and positively associated with immediate and delayed recall memory (statistically significant for affectionate support) [7].

*Cohort Studies*. Two studies of perceived support and memory found either no association [26] or statistically significant and positive associations with both working and episodic memory [60]. Liao and Scholes found a positive and statistically significant association between positive social support and global memory [84]. Hughes et al. found a negative association in the case of emotional support, and positive associations for instrumental support and satisfaction with social support [79]. Zahodne et al. found positive and negative associations, respectively, between emotional and instrumental support, and working memory; they also observed negative associations between emotional support and episodic memory, and no association between instrumental support and episodic memory [102].

##### Executive function

*Cross-Sectional Studies*. Gow et al. reported a positive and non-statistically significant association between participant satisfaction with functional social support and executive function, although they did not provide any numerical findings [34]. Hamalainen et al. and Krueger et al. reported positive and statistically significant associations between perceived social support and executive function [35, 43].

Three of four cross-sectional studies found positive associations between emotional social support and executive function, two of which were statistically significant. One study stratified by individual sources of emotional support and only spousal support remained statistically significantly associated with executive function. One study observed a statistically significant negative association [28, 31, 44, 59].

Three cross-sectional studies assessed the independent effect of instrumental social support on executive function: La Fleur and Salthouse found a small yet non-significant positive association, Zahodne et al. observed no association, and Bourne et al. reported a statistically significant negative association [28, 44, 59]. Ge et al. evaluated combined emotional and instrumental support on executive function and reported a statistically significant positive association [32].

*Cohort Studies*. Five cohort studies evaluated the effect of functional social support on executive function. Dickinson et al. and Liao & Scholes found positive and statistically significant associations for instrumental and positive support [74, 84]. Zahodne et al. showed a positive, but non-significant, association for emotional support and a negative, non-significant association for instrumental support [102]. Liao found no association for confiding support, and Hudetz et al. showed no significant association between perceived social support and post-operative executive function [26, 83].

##### Other cognitive domains

(Table 5). La Fleur and Salthouse’s cross-sectional study found a positive association between instrumental support and language ability, and a stronger and statistically significant association between emotional support and language ability [44]. Three cohort studies reported mixed results of no [26], positive [60], or negative associations (the latter being non-statistically significant) with language ability [102].

Two cross-sectional studies and one cohort study measured attention or processing speed. The cross-sectional studies reported positive associations for perceived social support [64, 65], with the former reporting a statistically significant result. The cohort study found no association for instrumental support, a positive association for emotional support, and a larger positive association with satisfaction with social support [79].

Three cross-sectional studies found slight protective effects between perceived/overall support and conversion to MCI [42, 52, 62]. One cohort study observed that negative social interaction was a risk for MCI [99].

## Discussion

Overall, functional social support was positively associated with cognitive function in middle- and older-aged adults (Fig. 3). However, the results were not uniform across the 85 included studies.

### Overall functional social support

Individual perceptions of functional social support did not appear to be associated with a diagnosis of AD or all-cause dementia. Conversely, perceived support was most often positively associated with improved cognitive function, although these associations did not always reach statistical significance. Negative associations, or a lack of association, were sometimes observed in the context of male participants or family members as the only sources of perceived social support [45, 70]. The negative association observed for male participants could suggest that males and females experience social support differently and emphasizes distinct aspects of the quality of social relationships. Social support from family members may be inversely associated with cognition because tumultuous intra-family relations could lead to psychosocial stress.

### Emotional social support

Most studies involving a clinical diagnosis of AD or all-cause dementia reported non-significant negative associations between emotional social support and these outcomes. Most of these studies also found significant and positive associations with both global and domain-specific cognitive function. However, negative associations or absence of any association were sometimes observed when considering emotional support provided by family members [79, 89]. Individuals in need of strong emotional support from their co-residing family members might concomitantly be experiencing some form of family-based physical or psychological stressors that negatively affect cognition.

### Instrumental social support

In contrast to the findings with perceived or emotional support, an equal number of studies observed positive and negative associations between instrumental support and AD or all-cause dementia. Most studies reported non-significant positive associations between instrumental support and domain-specific cognitive outcomes, although several studies in this group found an inverse association. For global cognitive function, an approximately equal number of studies reported positive and negative associations. The number of studies with negative associations was larger in the case of instrumental support compared to perceived and emotional support. Perhaps these findings merely reflect the increased need for functional support in day-to-day life among people with dementia, which can be partially provided by instrumental social support.

### Emotional-instrumental social support, satisfaction with social support

Most studies that assessed the combined effects of emotional and instrumental support reported positive associations with global and domain-specific cognitive function. All studies that assessed participant satisfaction with functional social support found protective effects against both AD and global dementia. All articles that measured domain-specific cognitive outcomes found satisfaction with social support to be non-significantly positively associated with cognition. Reported satisfaction with social support was also positively associated with global cognition in most cases.

### Positive, affectionate, confiding social support

Five studies examined positive, affectionate or confiding types of support [7, 51, 81, 83, 84]. Receiving positive social support was associated with a decreased risk of dementia, as well as improved global cognition and memory. Similarly, affectionate social support was associated with decreased risk of dementia and improved memory. One study explored the effects of confiding support on executive function and reported no association between the two variables.

### Domain-specific cognitive outcomes

Memory was the most frequently assessed, domain-specific cognitive outcome. In most cases, functional social support was positively associated with memory. The same results were found with executive function. Turning to the domains of language and attention/processing speed, all studies reported either no association or a positive association. Some studies used a clinical diagnosis of MCI as the cognitive outcome and found functional social support acted as a protective factor, whereas negative social interaction served as a risk factor.

### Strengths and limitations

A self-assessment with AMSTAR2 (Additional file 3) showed the quality of our systematic review was strong [103]. Our comprehensive search strategy captured many articles across a spectrum of functional social support exposures and cognitive outcomes. The nature of the exposure prevented us from looking at randomized controlled trials. One of the included articles was at high risk of bias and the narrative synthesis was facilitated by the similarity of covariate sets in the included articles.

Our review is unique from Kelly et al. [15] and Costa-Cordella et al. [19] because it focused exclusively on functional social support. Further, our review contained the most up-to-date synthesis of the literature on the topic. The adverse impact of the COVID-19 pandemic on social engagement, especially among older adults, provides a renewed impetus to understand how functional social support affects the cognitive health and well-being of aging populations.

## Conclusions

The findings of this review show that functional social support may act as a protective factor against dementia and cognitive decline. This association appears to be stronger in the case of overall and emotional support, relative to instrumental support. Policy makers may wish to allocate public funds for community-based programs centered on fostering quality social relationships high in emotional support among middle-aged and older adults.

## Supplementary Information

**Additional file 1.** PRISMA Checklist.

**Additional file 2.** Search strategy used in PubMed database.

**Additional file 3.** AMSTAR Checklist.

## Data Availability

The raw data extraction and risk of bias tables used during the current study are available from the corresponding author on reasonable request.

## Abbreviations

AD: Alzheimer’s disease
ADL: Activities of daily living
AMSTAR: A Measurement Tool to Assess Systematic Reviews
MCI: Mild cognitive impairment
NOS: Newcastle–Ottawa Scale
PRISMA: Preferred Reporting Items for Systematic Reviews and Meta Analyses
SWiM: Synthesis Without Meta-Analysis
