# Supplementary material for: Functional social support and cognitive function in middle- and older-aged adults: a systematic review of cross-sectional and cohort studies
Source: Syst Rev. 2023 May 22;12:86. doi: 10.1186/s13643-023-02251-z (PMC10200705; doi:10.1186/s13643-023-02251-z)
Supplement: Supplementary file 2 — Additional file 2. Search strategy used in PubMed database. [file 13643_2023_2251_MOESM2_ESM.docx]

**Additional file 2: Search strategy used in PubMed database**

**Outcome: Cognitive function**

Cognition[MeSH:noexp] OR Dementia[MeSH] OR “Alzheimer disease”[MeSH] OR “Cognitive dysfunction”[MeSH] OR “Executive function”[MeSH] OR “Neuropsychological tests”[MeSH] OR “Cognitive aging”[MeSH]OR "Intellectual functioning"[tiab] OR "Language processing"[tiab] OR "Visuospatial processing"[tiab] OR "Verbal learning"[tiab] OR "Processing speed"[tiab] OR Cognition[tiab] OR “Cognitive function”[tiab] OR “Cognitive functions”[ tiab] OR “Cognitive functioning”[tiab] OR “Memory”[tiab] OR “Cognitive ability”[tiab] OR “Cognitive abilities”[tiab]OR “Cognitive decline”[tiab] OR “Cognition disorders”[tiab] OR “Cognitive impairment”[tiab] OR “Cognitive impairments”[tiab] OR Dementia[tiab] OR “Alzheimer Disease”[tiab] OR “Alzheimer’s disease”[tiab] OR “Cognitive dysfunction”[tiab] OR “Cognitive dysfunctions ”[tiab] OR “Executive function”[tiab] OR“Executive functions”[tiab]OR“Cognitive aging”[tiab]OR “Cognitive ageing”[tiab]

**Exposure: Functional social support (and subtypes)**

"Social support survey" OR "Medical Outcome Study Social Support Survey" OR "MOS-SSS" OR "MOSSSS" OR "Functional social support" OR "Social support availability" OR "Perceived social support" OR "Social isolation"[ti] OR "Perceived social isolation" OR "Social support"[ti]OR “Social vulnerability”[ti] OR “Social resources”[tiab] OR “Emotional support”[tiab]OR “Emotional social support”[tiab]OR “Informational social support”[tiab]OR “Instrumental support”[tiab]OR “Instrumental social support”[tiab]OR “Tangible social support”[tiab]OR “Tangible support”[tiab]OR “Positive social interactions”[tiab]OR “Positive social support”[tiab]OR “negative social interactions”[tiab]OR “Affection social support”[tiab]OR “Affectionate support”[tiab]

**Age**

"Aged"[MesH] OR "Middle aged"[MesH] OR "Aged, 80 and over"[MesH] OR "Aging"[tiab] OR “Ageing”[tiab] OR “Older adult*”[tiab] OR “Older adults”[tiab]OR “Mid-life”[tiab] OR “Late-life”[tiab] OR “late life”[tiab] OR “Later life”[tiab]OR “Later-life”[tiab]OR “elderly”[tiab] OR “Cognitive aging”[tiab] OR “Cognitive ageing”[tiab]OR “Older”[TI]Language: English[lang] OR French[lang]
