# Supplementary figures and images for: Functional social support and cognitive function in middle- and older-aged adults: a systematic review of cross-sectional and cohort studies
Source: Syst Rev. 2023 May 22;12:86. doi: 10.1186/s13643-023-02251-z (PMC10200705; doi:10.1186/s13643-023-02251-z)

**Additional file 3: AMSTAR Checklist**
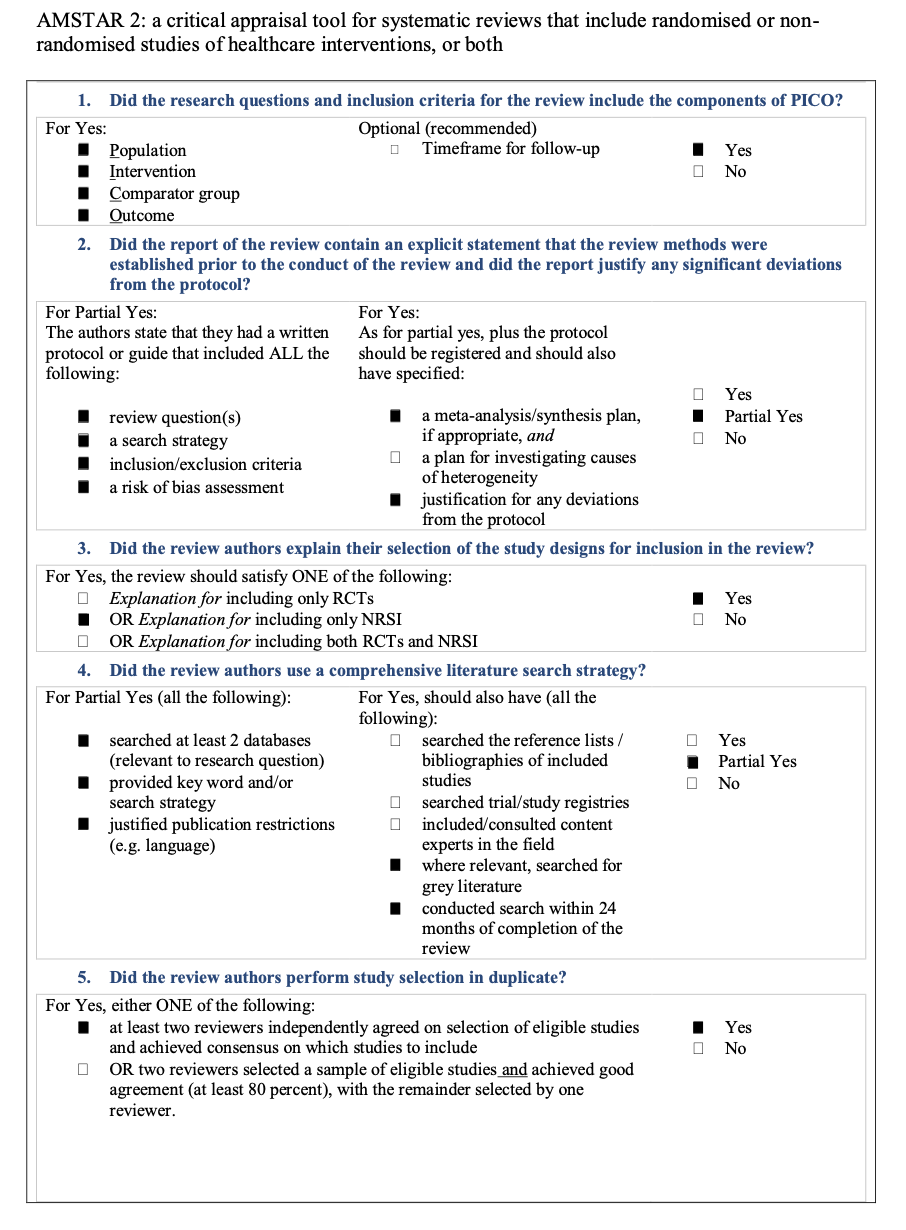

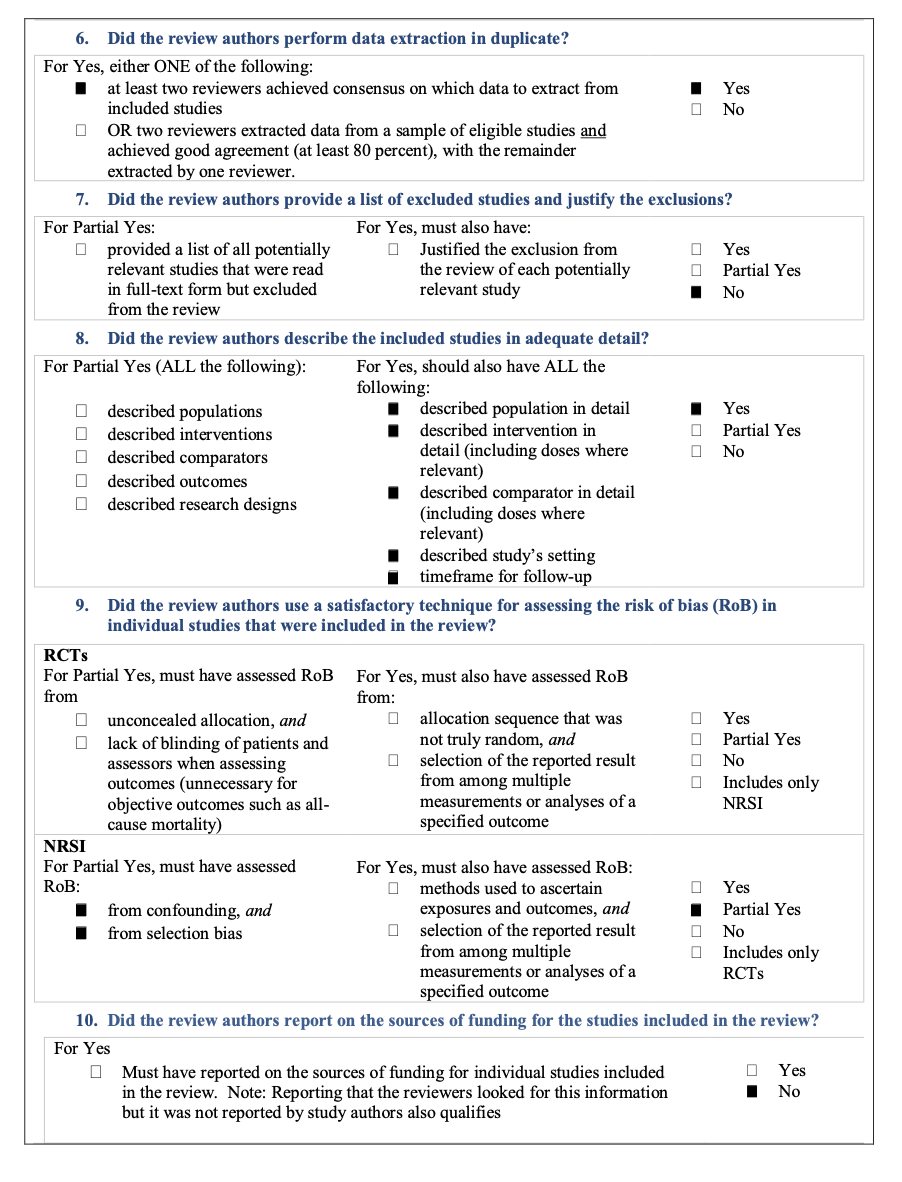

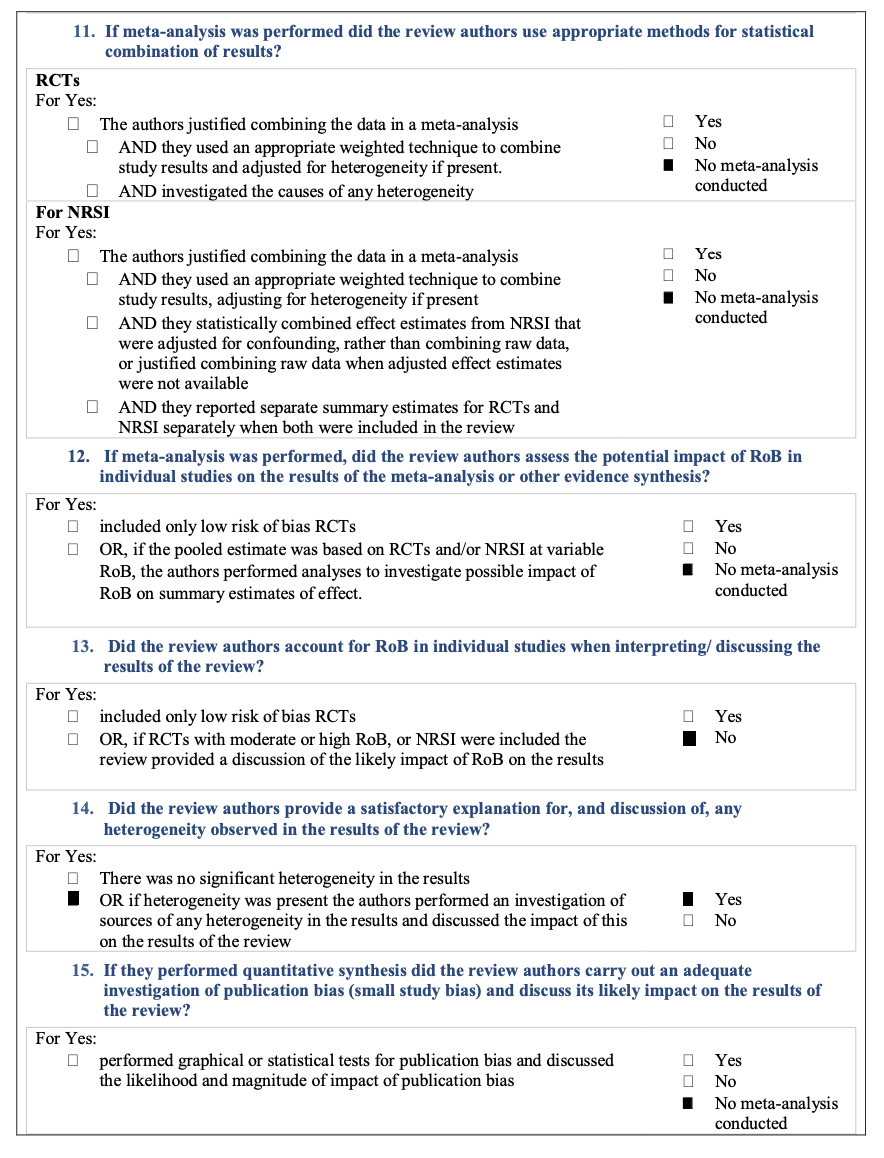

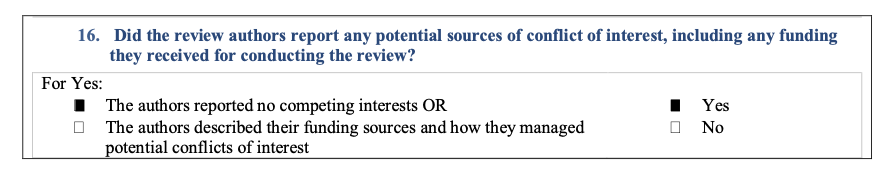

Supplement: Supplementary file 3 — Additional file 3. AMSTAR Checklist. [file 13643_2023_2251_MOESM3_ESM.docx]
